# Back from Exile? First Records of Chewing Lice (*Lutridia exilis*; Ischnocera; Mallophaga) in Growing Eurasian Otter (*Lutra lutra*) Populations from Northern Germany

**DOI:** 10.3390/pathogens12040587

**Published:** 2023-04-13

**Authors:** Simon Rohner, Joy Ometere Boyi, Valentina Artemeva, Olaf Zinke, Astrid Kiendl, Ursula Siebert, Kristina Lehnert

**Affiliations:** 1 Institute for Terrestrial and Aquatic Wildlife Research, University of Veterinary Medicine Hannover, Werftstrasse 6, 25761 Buesum, Germany; 2 Museum der Westlausitz Kamenz, 01717 Kamenz, Germany; 3 Aktion Fischotterschutz e.V., Otterzentrum Hankensbüttel, 29386 Hankensbüttel, Germany

**Keywords:** *Lutridia exilis*, eurasian otter, specialist parasites, population health, biodiversity

## Abstract

Arthropod ectoparasites of aquatic wildlife often have complex relationships with their host species that have developed over long evolutionary time scales. Specialist parasite occurrence might depend on these hosts’ distributions. Eurasian otter (*Lutra lutra*) populations are recovering in Northern German federal states, such as Schleswig-Holstein and Lower Saxony. Chewing lice (*Lutridia exilis*; Ischnocera; Mallophaga) are considered otter-specific yet rare parasites in their known range. In 2022, they were recorded for the first time on nine otters found dead in Northern Germany. All otters originated from the years 2021–2022 and were dissected during population health monitoring programs in 2022. Females (*n* = 6) were 0–5.5 years old and showed signs of disease in five cases. Males (*n* = 3), in contrast, were 0–1.6 years old and showed disease in a single case. Individual lice intensity of infection ranged from 1 to 75 specimens per otter. No direct adverse health effects of chewing lice on the otters were noted. *Lutridia exilis* morphological characteristics were documented and measurements were taken to study specialized adaptations that allow lice to attach to semi-aquatic otters. In addition, morphology was compared between lice from different geographical regions and specimens from previous reports. A region of the COI mDNA was amplified to molecularly characterize *L. exilis* for the first time and detect genetic differences between otter lice populations in Germany. It is believed that specialist parasites reduce in numbers even before their host populations decline. Recovering otter populations in Northern Germany could be an example of a reverse effect, where the comeback of a host species results in the return of a specialist parasite, which reflects an ultimate boost in overall species biodiversity.

## 1. Introduction

Public views on species biodiversity often focus on charismatic species, such as mammals, and neglect less attractive groups, such as invertebrates [1,2] and especially parasites, which are often perceived as gross [3,4]. This holds especially true for ectoparasites such as lice (Phthiraptera), which are generally very host specific and coevolve with their host species over long time periods [2,5,6], developing high levels of adaptation and specialization for their host and its environment [7,8,9]. Threatened and declining host populations with limited geographical ranges can therefore have cascading negative effects ontheir parasite communities [2,10,11,12]. There is evidence that certain parasites, which rely on direct transmission, might even become extinct before their hosts, when the host population levels drop below critical thresholds [1,13,14].

Indicating a present conservation story of success, Eurasian otters (*Lutra lutra*) are re-expanding their range in many Eastern and Northern German federal states [15,16,17,18,19,20,21]. Schleswig-Holstein (SH) is the northernmost federal state and acts as a linking bridge between German and Danish otter populations [17,19]. Today, otters can once again be found in most parts of Schleswig-Holstein [21]. Lower Saxony (LS) has not yet been completely recolonized by otters. According to the latest survey data, a rather isolated population exists in the south and only a few incidences of otter presence were found in the northwestern part of the federal state [18] (https://www.otterspotter.de/otterverbreitung, assessed on 26 January 2023). Benefiting from strict hunting bans in the former German Democratic Republic (DDR), otter populations started to recover in Saxony (S) earlier during the last century [22]. Representing the stronghold area of Germany’s otter population, most otter populations in other German federal states originate from the east [17]. Actual knowledge of the otter population health status is still scarce in Germany [21,23,24,25]. Despite the fact that most otters found dead in Germany are roadkill and are young and relatively healthy individuals, this is likely biased as diseased animals are less likely to be found [21,24]. To date, the few records of parasites from otters in Germany mainly represented ectoparasites, namely ticks [21,25,26]. In general, Eurasian otters reportedly were infected with *Ixodes ricinus*, *I. hexagonus*, *I. trianguliceps* and *I. canisuga* [25,26,27,28,29]. Various parasites are known to infect *Lutra lutra* in Europe, including zoonotic *Toxoplasma gondii*, where otters may represent accidental hosts [21,30,31]. Other documented endoparasites with probably low clinical relevance include *Isthmiophoramelis* [21,32,33] and *Sarcocystis lutrae*, the latter most likely infecting Eurasian otters as accidental hosts [21,34,35]. Different species of *Corynosoma* were found in Eurasian otters, and transient parasites from prey need to be taken into consideration [21,36,37]. Trematodes *Pseudoamphistumum truncatum* and *Metorchisalbidus* have infected Eurasian otters in the UK [38,39].

Chewing lice (*L. exilis*) [40] have been described as cryptic parasites, specialized to Eurasian otters [13,41,42]. Only occasional and dated lice records exist for Europe and Northern Africa [43,44,45], with regular sightings of these rare parasites in Eastern Germany since 1987 [43,44,45]. Here, otters survived in small but stable numbers over the last century, when most of Germany’s other otter populations were eradicated [15,20]. Chewing lice (Ischnocera; Mallophaga) usually feed on the skin and skin products of their mammal hosts [46], or feathers in birds, which they supposedly metabolize with the aid of endosymbiotic bacteria and are usually host specific, only found on one genus or species of host. Transmission occurs via direct body contact. An individual louse lays eggs from which three nymphal stages hatch before becoming mature. Although perceived as relatively benign parasites and often found in mild infection levels that are regulated by host grooming in healthy individuals, they can cause skin irritations and reduced fitness when occurring at higher intensities [45]. In addition, chewing lice can serve as vectors for filarial disease [47]. Parasites are considered important bio-indicators of host ecology and can reflect ecosystem traits and highlight population dynamics [48,49,50,51]. To understand the role of the tiny and elusive parasites infecting mobile wildlife species such as otters, morphological investigations using light microscopy and high-resolution imaging for identification are ideally complemented by molecular techniques to unequivocally determine the species [50,52]. The aim of this study is to identify ectoparasitic chewing lice on Eurasian otters found for the first time in Northern Germany and characterize the rare and specialized parasite *L. exilis* using morphological and molecular tools. The importance and impact of its reappearance in regard to its host health, ecology and overall biodiversity changes are discussed. Recolonizing otters in Northern Germany may contribute to an overall increase in biodiversity by their return together with their parasite fauna, which can highlight their geographic origin.

## 2. Materials and Methods

### 2.1. Carcass Collection

Since 2019, otters found dead in SH are routinely collected and examined in the frame of a program funded by the Ministry for Energy Transition, Protection of the Climate, Environment and Nature (MEKUN) that aims at establishing the long-term monitoring of the species [21]. Otters from LS are collected and stored opportunistically on a voluntary basis by the Aktion Fischotterschutz e.V., Otter-Zentrum Hankensbüttel, a non-governmental organization and specialized zoo for mustelids. All nine animals were necropsied at the Institute for Terrestrial and Aquatic Wildlife Research (ITAW) in Büsum, Germany, according to [21]. In total, four carcasses were dissected in a fresh condition, three were cooled at +4 °C before dissection and two otters were stored frozen at −20 °C and thawed before dissection. One free-ranging otter from LS was taken into rehabilitation in 2021, where it died six months later. The date of finding in this study represents the actual date of death for this individual.

### 2.2. Dissection

A special otter dissection protocol was used for all nine otters in the frame of this study [21]. All otters were weighed and measured, including total body length and girth. Based on the subcutaneous fat depots in the tail radix region, intra-abdominal fat storage and the development of skeletal muscles along the spine, the nutritional state was assessed, ranging from good to moderate to poor. Decomposition status ranged from 1 (i.e., fresh) to2 (i.e., good) to 3 (i.e., moderate) to 4 (i.e., progressed decomposition) to 5 (i.e., severe decomposition).

### 2.3. Age Determination

A lower or upper canine was taken during dissection for cementum aging [21]. Counting of incremental layers and age interpretation followed [53].

### 2.4. Parasitology

Each otter was checked for ectoparasites macroscopically and also by using a lice comb on the head, in the axillar and anogenital region. Parasites were further counted to determine the intensity of infection [54]. Arthropod ectoparasites were morphologically identified using a light microscope (Olympus SZ61) based on the descriptions of [40,55,56]. The encountered insect ectoparasite individuals were sexed and divided into mature males, mature females, nymphs and nits.

Additional chewing lice individuals collected in ethanol (*n* = 17; originating from two otter hosts) and dried (*n* = 231; originating from 26 otter hosts) by a health monitoring program of otters in Saxony were counted, measured and submitted for genetic analysis and imaging techniques for comparison after rehydrating the dried specimens in 70% ethanol.

All collected insect ectoparasites from infected otters from Schleswig-Holstein, Lower Saxony and Saxony were measured for six indices using CellSensEntry V3.2 software (Olympus, Hamburg, Germany) and the measurement program ImageJ (Version 1.53k) with a stereomicroscope (Olympus SZX10) with 100× magnification and attached camera (Olympus UC90). The total length was measured from the anterior margin of the head to the distal part of the last segment of the abdomen (posterior margin of the genital apparatus or segment IX in males [44,55] and the width of the head from the right to left lateral margin of the temple [56]). Head length was measured dorsally from the anterior margin of the head to the caudal margin of the back of the head, the occipital ring [56]. Widths of the prothorax and mesometathorax were measured on the dorsal side of the specimens and the abdomen width was measured at its widest point (segment 3–4). Nit total length was measured from the anterior margin of the operculum to the posterior margin of the nit body, whereas the width was measured between the two most distant lateral points of the nit, perpendicular to the axis of the nit [57]. Measurements were compared between different geographic regions and additionally to previous historic and dated national publications in German language reporting on otter ectoparasite morphology [43,44,57,58].

For documenting specimens at the Center of Natural History of the Zoological Museum Hamburg (CeNak), all developmental ectoparasite stages were photographed using the BK Plus Lab System (Dun, Inc., Jacksonville, FL, USA) with 5× and 10× LD Mitutoyo objectives and integrated Canon (Canon 7D Mark II (20 Megapixels)) Micro camera and Canon 5DS (50 Megapixels) Macro camera. All images were captured and stacked with the Zerene Stacker software version 1.04.

### 2.5. Molecular Parasite Identification and Phylogenetics

Genomic DNA was isolated from at least one insect ectoparasite specimen from seven otter individuals found in the German federal states of Schleswig Holstein (*n* = 5) and Lower Saxony (*n* = 2) using the QIAamp DNA Micro kit (Qiagen, Hilden, Germany). A 710bp region of the mitochondrial gene encoding cytochrome c oxidase subunit 1 (COI) of these samples was amplified using the primers LCO1490 (GGTCAACAAATCATAAAGATATTGG) and HCO02198 (TAAACTTCAGGGTGACCAAAAAATCA) [59]. The polymerase chain reaction (PCR) products obtained were Sanger-sequenced in both directions. The sequences were examined in SnapGene Viewer 5.3.2 and compared to available Trichodectidae sequences on GenBank using BLASTN.

For comparative analyses, sequences from ectoparasite specimens were obtained from dead otters from Saxony (S) in Eastern Germany (*n* = 4) (Table 1). For phylogenetic analyses, available sequences of carnivoran trichodectid species (*n* = 3) were downloaded from GenBank (MT027226, AF545736, AF545700). Sequences were aligned using MAFFT [60], and *Ricinu smarginatus* and *Ricinus elongatus* were designated as outgroups. GBlocks version 0.91b [61] was used to cut significant gaps in the resulting alignment (default parameters, allowed gap position—“with half”). A maximum likelihood phylogenetic tree with 500 bootstrap replicates was constructed in MEGA X software [62] using the GTR+G model selected based on Akaike Information Criterion (AIC) in jModeltest2 v 0.1.11 [63].

### 2.6. Statistical Analysis

All statistical analyses were performed in R software (version 4.2.1). Data visualization was performed using the ggplot2 package. A Mann–Whitney U test was used to check for differences between locations (Northern Germany and Saxony) and method of preservation (samples stored in ethanol vs. dried samples). The significant level was set at α = 0.05.

## 3. Results

### 3.1. Otter Life History and Dissection Results

**Figure 1:**
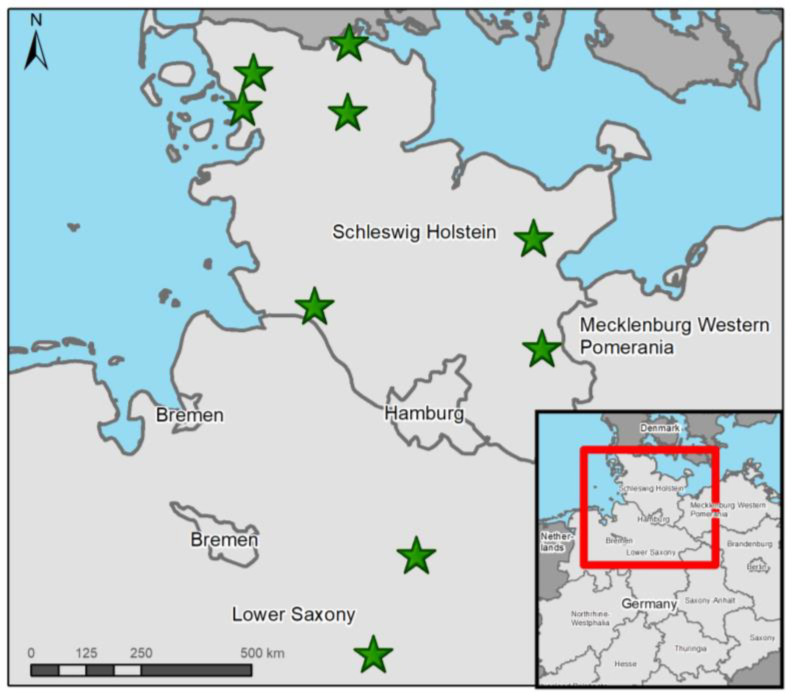
Finding locations (green stars) of all otters from Schleswig-Holstein and Lower Saxony investigated in this study. Map made with ArcGis 10.8.2 (© ESRI, Inc., Redlands, CA, USA).

All otter individuals from SH originated from 2022, except one otter which was from 2021 (Table 2). In LS, one otter was found in each year for 2021 and 2022 (Figure 1). The months in which otters were found included January (*n* = 1), February (*n* = 1), April (*n* = 3), August (*n* = 2), October (*n* = 1) and December (*n* = 1). Otter no. 1 was taken into rehabilitation in April 2021, were it later died. Otter no. 6 was an orphaned cub that died in veterinary care. Age ranged from 0 to 5.5 years; notably, three investigated otters were cubs. Most otters were juveniles or young adults, ranging from 0.6 to3.1 years. More females (*n* = 6) compared to males (*n* = 3) were investigated in this study. Besides four road-killed otters, five died due to various causes and displayed symptoms of clinical disease. Of those five, two individuals succumbed to clinical disease. Otters no. 5 and 6 were diseased but ultimately starved, and otter no. 7 was euthanized due to serious gross lesions but also carried bone fractures resembling traumatic injury, typically caused by vehicle collision. Otter no. 9 did show signs of disease; however, this probably did not significantly impair its health status. Road accidents were the cause of death of four individuals. Only three otters were in good nutritional status, while two were in moderate and four were in bad body condition. Decomposition grade ranged from 1 (*n* = 2) to 2 (*n* = 3) to 3 (*n* = 2) to 4 (*n* = 2). The majority of otters were dissected fresh (*n* = 4) or cooled (*n* = 3), and only two individuals were frozen and thawed before necropsy.

### 3.2. Chewing Lice and Nits

**Figure 2:**
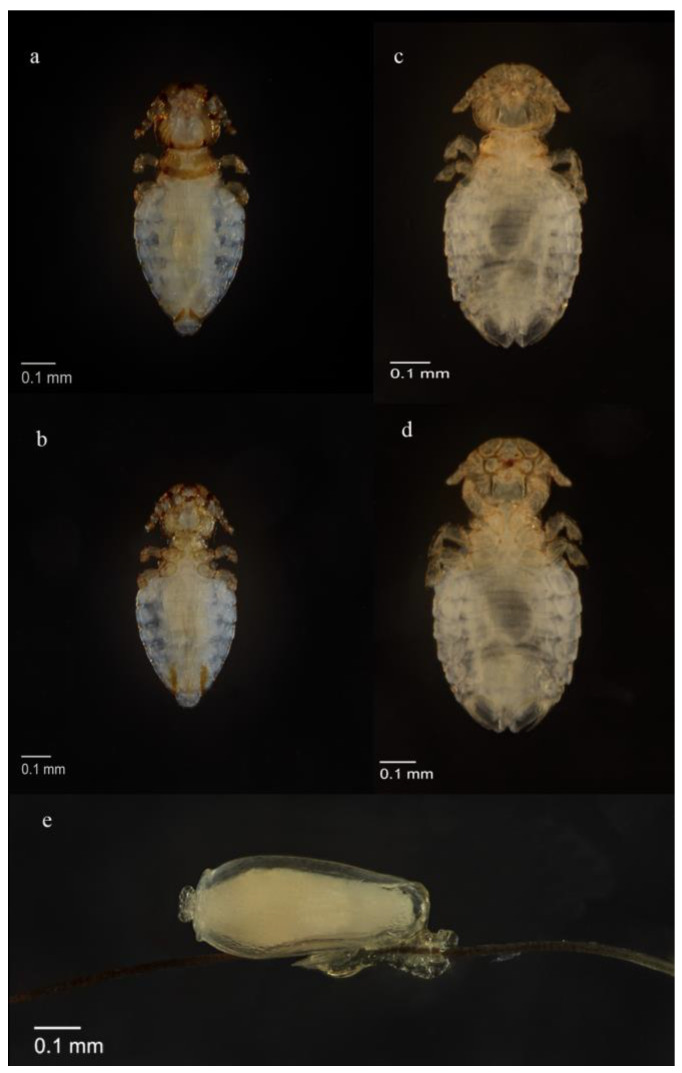
(**a**) *Lutridia exilis* adult male dorsal view; (**b**) *Lutridia exilis* adult male ventral view showing genitalia; (**c**) *Lutridia exilis* adult female dorsal view, (**d**) adult female ventral view, (**e**) nit attached to hair; all sampled from Eurasian otters (*Lutra lutra*) in Germany.

All arthropod ectoparasites were found in the head region on the nine infected otters. Most investigated otters carried only a few specimens, apart from one individual, from which 75 parasite specimens were recovered. The observed parasite individuals had a louse-like appearance and were minute, wingless, dorsoventrally flattened insects with their bodies divided into a head, prothorax, mesometathorax and an abdomen with nine segments. According to their morphological characteristics, they were identified as *Lutridia exilis* (Insecta; Phthiraptera), a trichodectid parasitic chewing louse of otters. Males were characterized by a round protruding copulative organ on their last abdominal segment (Figure 2a,b). Females were distinguished by the presence of two copulation valves (gonopods) positioned caudoventrally (Figure 2c,d) and a larger and more rounded abdomen in contrast to males. Nymphs had no distinguishable sex organ and were smaller than mature individuals. Nits were egg-shaped, consisting of the nit body, its operculum cap with aeropyles for ventilation and cementum, with which they were attached to the hair of the hosts (Figure 2c,d).

Of the 96 *Lutridia exilis* lice recovered from the Northern German otters, 48% (*n* = 46) were females, 23% (*n* = 22) males, 16.5% nymphs (*n* = 16) and 12.5% nits (*n* = 12). Females measured between 0.94 and 1.25 mm in length and 0.42 and 0.66 mm in width and were significantly (*p* < 0.05) larger than males, which measured between 0.81 and 1.02 mm in length and 0.27 and 0.50 mm in width. Nymphs were 0.70–0.90 mm in length and 0.40–0.54 mm in width and nits ranged from 0.9 to 1.0 mm in length and from 0.4 to 0.6 mm in width (Table 3).

No difference in size was observed between dried specimens rehydrated with 70% ethanol for the analyses and ethanol-preserved specimens (*p* > 0.05); however, otter lice from Northern Germany were significantly larger than those from Saxony (*p* < 0.05). The measurements of chewing lice found in this study were in the same range as those of previous studies (Table 4).

### 3.3. Molecular Characterization

A 600 bp-long COI sequence was obtained from eight chewing lice specimens originating from six otter individuals from SH and one individual from LS. The comparative sequences of otters from Saxony were derived from five specimens from four otter individuals. The chewing lice sequences were 75% identical to *Trichodectes canis* (MT027226.1) available on GenBank. Sequences of chewing lice from otters in SH and LS (Northern Germany) were 99–100% identical. However, the COI sequences derived from chewing lice from otters in Saxony differed by 6%, comprising fourteen single nucleotide polymorphisms from chewing lice originating from Northern Germany, except the sample collected in 2021, which was 99% identical to samples from Northern Germany.

**Figure 3:**
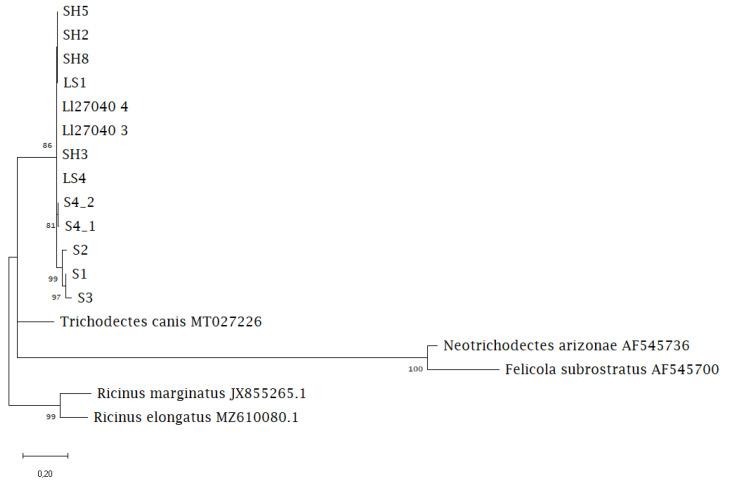
Maximum likelihood phylogenetic tree showing the relationship between *Lutridia exilis* sequences and Trichodectidae sequences from carnivoran hosts available on GenBank.

Phylogenetic analysis revealed a monophyletic *Lutridia exilis* separate from the other Trichodectidae species. The sequences of chewing lice from Saxony were placed as a sister group to the sequences from Northern Germany, with one of the Saxony sequences within the Northern Germany group. *Trichodectes canis* from dogs and *Neotrichodectes arizonae* from hog-nosed skunks were clustered together as sister taxa (Figure 3).

## 4. Discussion

Chewing lice *Lutridia exilis* were reported for the first time on otters in Northern Germany. Except for two otters that originated from 2021, all other studied animals were found in 2022. Consequently, the emergence of chewing lice in Northern Germany needs to be dated back to 2021 at least. Although they have been reported from the south and east of Germany before [44], their occurrence in the last century was a rare event in the overall known distribution range. Thus, their appearance on otters in SH and LS indicates the re-extending distribution range of the otter populations and, consequently, the spread of their parasite communities. So far, only low numbers of chewing lice have been recorded on Eurasian otters in Northern Germany, which may be due to the relatively recent return of otters to the study area [19,21]; therefore, an inference on prevalence remains difficult. Although chewing lice are commonly reported as occurring in low intensities [41,43,44], a single reported infection with approximately 1000 specimens on one otter is known [45]. Mostly mild intensities of infection (1–75 lice per otter) were observed in this study, which may also be due to the loss of ectoparasites postmortem. Ectoparasites were reported to leave their dead host soon after death and can be lost during transport of the carcass and before dissection, consequently introducing a bias in prevalence and intensity observed, as also discussed in other post-mortem studies investigating the ectoparasites of otters [26,29]. However, the majority of otters used in this study were dissected fresh or after initial cooling, meaning some individuals were stored at +4 °C overnight until examination. In addition, chewing lice were recovered from two code four decomposition grade otters, indicating that the parasites might stay on their hosts even days after their death. Therefore, the observed infection rates are likely to at least partially reflect the initial infection rates of the otters before death.

All chewing lice were detected in the area of the head of the investigated otters, supporting previous findings [45]. Because the initial finding of a chewing louse on an otter from SH occurred rather by chance during a routine sampling process at dissection, the question remains whether lice were simply overlooked in the past. This, however, did not seem very likely as macroscopical ectoparasite screening, also with the help of a lice comb, was conducted from the beginning of the post-mortem investigations of otters in 2019, and most otters were dissected by the same veterinarian [21]. Interestingly, three out of the nine studied otters were abandoned cubs. Two were roadkill in good body condition, while the third one had starved and also displayed signs of clinical disease. As health did not seem to be a critical factor for lice infection among these cubs, the close contact of the mother with the cubs could play an important role in lice transmission in this age class. Whether the transmission of parasites might be favored in the dens of the females can only be speculated at this point. Among the six subadult to adult otters, two were roadkill in good and moderate body condition. The other four otters displayed serious symptoms of clinical disease and the body condition was moderate in one and bad in three cases. Notably, one out of the four otters was euthanized in veterinary care due to its serious condition. At necropsy, fractures of the skull indicated this individual might have experienced a traumatic injury, such as a vehicle collision, before it was found. Consequently, it might have starved due to the inability to catch prey. Interestingly, the majority of the investigated otters in this study were diseased, which is contrary to the general population health status observed for otters in Northern Germany [21]. Although the infection with chewing lice did not seem to have seriously affected the health of the studied otters here, there are indications that immunocompromised otters may be more susceptible to lice infections [45]. In the future, special attention should be paid to the histopathological investigations of chewing lice-infected skin areas in otters to better determine the health consequences of lice infections on their hosts.

When analyzing the historic chewing lice literature for morphological identification, different details in drawings depicting *Lutridia exilis* became apparent. *Lutridia exilis* drawings show an outward curve on the distal end of the gonopods in females in some figures by [57]. This was not observed in other specimen drawings [57], and lice from this study. However, specimens identified as *Lutridia matschiei* were drawn with a rounded shape of the gonopods in females [57] similar to the chewing lice found in this study. These differences may be due to morphological variation or could be mounting artefacts, but when some authors refer to drawings of previous publications [57], it is not clear if they reflect distinct species or potentially synonyms in the early literature. The significant differences in size measurements between chewing lice from Saxony and specimens from SH and LS indicated that a geographic separation between otter populations may have resulted in a differentiation between their parasite species. Nonexistent differences in size measurements between dried specimens that were rehydrated for analyses and specimens preserved in 70% ethanol from the same geographic location (Saxony) highlighted that archived material from both dried and wet parasite sample material is comparable and compatible for morphological investigations.

To support morphological species identification, for the first time, nucleotide data from the mtDNA COI loci was analyzed, and sequences derived from different geographical areas were compared. The results revealed genetic differences between otter chewing lice from the eastern and northern part of Germany, supporting the morphological differences observed in the parasites. The observed molecular differences were consistent among regions and indicated intraspecific variation. It is therefore assumed that a spatial segregation of otter populations in Germany resulted in phylogenetic changes in chewing lice. It remains striking that the COI sequence of a chewing louse specimen from a recently sampled otter (2021) in Saxony was similar to the Northern Germany chewing lice sequences, and did not display the 6% nucleotide difference observed in the previous specimens. The dispersion of otters within increasing populations and less segregation between areas, resulting in connected populations, may be responsible for this. Trichodectid chewing lice are predominantly associated with carnivores and especially with Mustelidae [64], therefore are believed to have a long phylogenetic relationship with their mammalian hosts [46]. Little is known about the biology and life history traits of the rare ectoparasite *Lutridia exilis*, which is why further research on these rare parasites is urgently needed. *Lutridia exilis* chewing lice are specialized insects that have highly adapted to their semiaquatic hosts. Specialized parasites are believed to undergo prolonged coevolution with their hosts, although at slower evolution rates, and thus are especially vulnerable to becoming extinct, similarly to their hosts [45,46]. However, avian lice were shown to have higher mtDNA evolution rates than their hosts and other insects [65]. Whether this applies to otter chewing lice, and whether small effective population sizes of *L. exilis* and transmission strategies influence this, remains to be shown. Consequently, they represent important components of species biodiversity [9]. Continuous post-mortem investigations are needed to further shed light on how increasing otter populations influence the density-dependent infection patterns of these directly transmitted ectoparasites. So far, it can only be speculated that the returning otters and their chewing lice constitute a healthier ecosystem that is richer in parasites than before [51,66]. Increasing biodiversity may increase the density of competent hosts for parasites, which may amplify parasite abundance [9,67]. However, on the contrary, increasing biodiversity could theoretically cause a dilution effect by reducing the abundance of a parasite species per host and the resulting infectious disease risk [68].

## 5. Conclusions

It is often forgotten that charismatic host species, recovering from former population declines, influence biodiversity in their ecosystems also by adding their parasite communities [8]. Chewing lice *Lutridia exilis* appearing in recovering otter populations in Germany could be the first sign of parasite communities bouncing back after decades of absence. Morphological and genetic analyses suggested that the past geographic separation of otter populations in Germany resulted in the differentiation of parasites. More research is needed to fully understand the adaptations and potential health effects of chewing lice on otters. Besides archived specimens from museums or biobanks, post-mortem investigations of wildlife, such as otters, offer unique opportunities to sample parasites from their hosts and collect vast amounts of data over long-term periods and highlight environmental and ecosystem changes.

## Figures and Tables

**Table 1:** Chewing lice specimens from otters originating from Saxony used for molecular analysis.

No. Otter	Finding Year	Federal State
S1	2017	Saxony
S2	2017	Saxony
S3	2019	Saxony
S4	2021	Saxony

**Table 2:** Life history and health data of all necropsied otters.

No. Otter	Finding Date	Federal State	Sex	Age (Years)	Decomposition Status	Preparation	Nutritional Status	Cause of Death	No. of Collected Lice per Host (Intensity of Infection)
LS1	7 October 2021	LS	f	0.8–2.25	2	frozen	bad	Disease	1 ♂
SH2	14 December 2021	SH	f	0	3	cooled	good	Roadkill	1 ♀
SH3	17 January 2022	SH	f	0.6–2.5	1	fresh	moderate	Disease	2 ♀, 6 nits
LS4	25 February 2022	LS	m	0.7–1.6	4	frozen	moderate	Roadkill	1 ♀
SH5	2 April 2022	SH	f	4.8–5.5	4	cooled	bad	Starving; Disease	1 ♀
SH6	12 April 2022	SH	m	0	1	fresh	bad	Starving; Disease	2 ♀, 5 nits
SH7	13 August 2022	SH	f	1.2–3.1	2	fresh	bad	Euthanasia; susp. Roadkill; Disease	1 nit
SH8	31 August 2022	SH	m	0.4–1.1	3	cooled	good	Roadkill	1 ♀
SH9	23 October 2022	SH	f	0	2	fresh	good	Roadkill; suspected disease	38 ♀, 21 ♂, 16 nymphs

LS: Lower Saxony; SH: Schleswig Holstein.

**Table 3:** Measurements (in mm) of chewing lice *Lutridia exilis* from Northern Germany (*n* = 96) and Saxony preserved in 70% ethanol (*n* = 17) and rehydrated in 70% ethanol after preservation as dried specimens (*n* = 231).

	Total Length		Head Length		Head Width		Head Index		Prothorax Width		Mesometathorax Width		Abdomen Width	
	Min–Max	Mean ± SD	Min–Max	Mean ± SD	Min–Max	Mean ± SD	Min–Max	Mean ± SD	Min–Max	Mean ± SD	Min–Max	Mean ± SD	Min–Max	Mean ± SD
Northern Germany (Females) (*n* = 46)	0.94–1.25	1.16 ± 0.06	0.24–0.29	0.26 ± 0.01	0.26–0.33	0.30 ± 0.01	0.81–1.0	0.88 ± 0.04	0.21–0.26	0.24 ± 0.01	0.25–0.34	0.31 ± 0.02	0.42–0.66	0.59 ± 0.04
Northern Germany (Males) (*n* = 22)	0.81–1.02	0.94 ± 0.05	0.23–0.28	0.26 ± 0.01	0.25–0.29	0.26 ± 0.01	0.89–1.04	0.97 ± 0.04	0.21–0.23	0.22 ± 0.01	0.25–0.29	0.27 ± 0.01	0.27–0.50	0.45 ± 0.05
Northern Germany (Nymphs) (*n* = 16)	0.71–0.99	0.90 ± 0.07	0.21–0.29	0.23 ± 0.09	0.23–0.29	0.26 ± 0.01	0.83–0.97	0.89 ± 0.04	0.19–0.25	0.23 ± 0.01	0.22–0.30	0.28 ± 0.02	0.40–0.54	0.47 ± 0.04
Northern Germany (Nits) (*n* = 12)	0.87–1.01	0.92 ± 0.04											0.44–0.57	0.50 ± 0.04
Saxony Ethanol (Females) (*n* = 5)	0.80–1.05	0.93 ± 0.08											0.42–0.52	0.47 ± 0.05
Saxony Ethanol (Males) (*n* = 4)	0.82–0.90	0.87 ± 0.03											0.34–0.43	0.39 ± 0.04
Saxony Ethanol (Nymphs) (*n* = 8)	0.55–1.01	0.81 ± 0.16											0.29–0.47	0.39 ± 0.06
Saxony Dry (Females) (*n* = 71)	0.86–1.14	0.99 ± 0.07	0.24–0.29	0.26 ± 0.01	0.27–0.33	0.30 ± 0.01	0.77–0.97	0.87 ± 0.04	0.18–0.31	0.24 ± 0.02	0.24–0.34	0.31 ± 0.02	0.30–0.63	0.54 ± 0.04
Saxony Dry (Males) (*n* = 70)	0.68–0.94	0.79 ± 0.06	0.23–0.28	0.25 ± 0.01	0.22–0.31	0.26 ± 0.01	0.82–1.05	0.95 ± 0.04	0.16–0.24	0.22 ± 0.01	0.21–0.30	0.26 ± 0.02	0.37–0.48	0.42 ± 0.02
Saxony Dry (Nymphs) (*n* = 90)	0.43–1.08	0.72 ± 0.14	0.16–0.29	0.22 ± 0.02	0.19–0.32	0.26 ± 0.02	0.76–1.02	0.88 ± 0.06	0.16–0.27	0.22 ± 0.02	0.18–0.32	0.26 ± 0.03	0.28–0.56	0.42 ± 0.06

**Table 4:** Measurements (in mm) of *Lutridia exilis* from Eurasian otters (*Lutra lutra*) in this study in comparison with previous studies.

	Length		Width	
	Females	Males	Females	Males
Conci, 1940	0.98–1.20	0.8–1.0		
Werneck, 1948	1.19	0.9		
Mey and Stubbe, 1989	1.03–1.19	0.89	0.54–0.61	0.42
Mey and Zinke, 1992	0.96–1.02		0.49–0.58	
Northern Germany, 2022 (this study)	0.94–1.25	0.81–1.02	0.42–0.66	0.27–0.50
Saxony Ethanol, 2022 (this study)	0.8–1.05	0.82–0.90	0.42–0.52	0.34–0.43
Saxony Dry, 2022 (this study)	0.86–1.14	0.68–0.94	0.30–0.63	0.37–0.48

## Data Availability

Not applicable.
